# Development of a scoring system to predict endovascular crossing of femoropopliteal artery chronic total occlusions: the Endo VAscular CROSsing Score for Chronic Total Occlusions (EVACROSS-CTO)

**DOI:** 10.1093/bjr/tqaf004

**Published:** 2025-01-15

**Authors:** Nikolas Matthaiou, Michail E Klontzas, Konstantina Kasioumi, George A Kakkos, Elias Kehagias, Dimitrios Tsetis

**Affiliations:** Department of Radiology, School of Medicine, University of Crete, Voutes Campus, Heraklion, Crete 71110, Greece; Department of Radiology, School of Medicine, University of Crete, Voutes Campus, Heraklion, Crete 71110, Greece; Interventional Radiology Unit, Department of Medical Imaging, University Hospital of Heraklion, Heraklion, Crete 71110, Greece; Interventional Radiology Unit, Department of Medical Imaging, University Hospital of Heraklion, Heraklion, Crete 71110, Greece; Interventional Radiology Unit, Department of Medical Imaging, University Hospital of Heraklion, Heraklion, Crete 71110, Greece; Department of Radiology, School of Medicine, University of Crete, Voutes Campus, Heraklion, Crete 71110, Greece; Interventional Radiology Unit, Department of Medical Imaging, University Hospital of Heraklion, Heraklion, Crete 71110, Greece; Department of Radiology, School of Medicine, University of Crete, Voutes Campus, Heraklion, Crete 71110, Greece; Interventional Radiology Unit, Department of Medical Imaging, University Hospital of Heraklion, Heraklion, Crete 71110, Greece

**Keywords:** score, chronic total occlusion, CT, endovascular, peripheral artery

## Abstract

**Objectives:**

To develop a predictive score for the prediction of successful endovascular crossing in femoropopliteal artery chronic total occlusions (CTOs).

**Methods:**

In this retrospective study, 84 patients were divided 70%:30% into a training and a testing cohort. Parameters such as cap morphology, side branches, bridging collaterals, flush occlusion, and length were derived from pre-procedural CT angiography. Lesions were segmented and calcification burden was assessed by thresholding. A score (EndoVAscular CROSsing Score for Chronic Total Occlusion [EVACROSS-CTO]) was built based on multivariate logistic regression. Receiver operating characteristics (ROC) curve analysis determined the optimal score threshold, with reported accuracy, sensitivity, specificity, and area under the curve (AUC).

**Results:**

Factors including age > 50 years (*P* = .036, odds ratio (OR) = 53.7), calcification percentage >10% (*P* = .011, OR = 16.63), the presence of a flush occlusion (*P* = .02, OR = 15.564), the presence of a distal side branch (*P* = .018, OR = 9.879), and the presence of a proximal side branch (*P* = 0.064, OR = 23.369) were identified as suitable for inclusion in the score. Score values were assigned based on the relative odds ratio for each factor with a maximum score of 22. EVACROSS-CTO was able to predict the success of endovascular recanalization with an AUC-ROC of 79.8% (95% CI, 58.5%-100%). A score >16 yielded a sensitivity of 75% with a specificity of 70.6% for the prediction of treatment failure.

**Conclusions:**

A score was developed by incorporating variables derived from pre-procedural CT angiography, demonstrating promising predictive capacity in determining the success of endovascular recanalization of CTOs.

**Advances in knowledge:**

EVACROSS-CTO incorporates imaging variables for the prediction of endovascular recanalization success. This score will allow improved pre-procedural planning for femoropopliteal CTO management.

## Introduction

Chronic total occlusions (CTOs) of infrainguinal arteries are frequently observed in patients with peripheral arterial disease (PAD). They are often associated with high rates of treatment failure and complications during endovascular treatment (EVT).^1^ Chronic total occlusions have posed significant challenges due to various factors affecting successful lesion crossing. Factors like calcification burden, lesion length, collateral circulation, and cap morphology are considered some of the most critical determinants, and extensive research has aimed to establish their correlation with recanalization success.^2–5^ Several advanced techniques have been developed to enhance technical success, including STAR, CART, and SAFARI, complemented by cutting-edge equipment, such as re-entry catheters and CTO-dedicated guidewires.^6^

Recently, there has been a growing interest in the development of scoring systems aimed at predicting the likelihood of successful crossing in cases of CTOs within coronary and peripheral arteries. Notably, 2 distinct predictive tools, specifically designed for below-the-knee (BTK) occlusions, have been proposed. These tools provide clinicians with objective criteria aiding them in selecting the optimal crossing approach, based on the angiographic evaluation.^3^^,^^7^ However, it is worth noting that a comparable predictive tool targeting femoropopliteal CTOs, utilizing pre-procedural data such as CT angiography or MR angiography, has yet to be proposed. The potential impact of such a tool on clinical decision-making is considerable, as it could contribute to the determination of whether EVT or surgical bypass is the more suitable option. Furthermore, it could assist in the selection of specific equipment and the application of appropriate endovascular techniques, enhancing the precision and success of interventions in this specific anatomical context, thereby improving patient outcomes and resource allocation.

The aim of this manuscript was to develop and test a predictive score that can be used in everyday clinical practice to predict successful endovascular crossing of peripheral artery CTOs.

## Methods

### Patient recruitment

In this retrospective observational descriptive single-centre study, all consecutive patients meeting specific inclusion criteria were enrolled: (1) symptomatic atherosclerotic PAD patients undergoing EVT and (2) CT angiography depicting a CTO in superficial femoral artery (SFA) and/or popliteal artery (POPA). Exclusion criteria were (1) patients without pre-operative CTA, (2) lesions extending below the trifurcation, and (3) prior endovascular intervention in the same lesion.

A total of 139 patients with CTOs were retrospectively recruited, but ultimately, 84 patients (68 male-16 female) met the inclusion criteria and were incorporated into this study. The remaining patients were excluded from further analysis due to not meeting the specified exclusion criteria. The patient data were retrieved from the hospital’s electronic database spanning the period between 2016 and 2022, encompassing details regarding the procedures performed and pre-procedural imaging assessments.

The dataset was split 70%:30% in cohort A used to construct the score and cohort B to test the score. The initial 59 consecutive patients (cohort A) were used to build the score, while the subsequent 25 consecutive patients (cohort B) were used to assess the predictive capacity of the score. The study has received approval from the Ethical Review Board of our University Hospital and all patients have signed an informed consent to undergo the procedure.

### Imaging and procedural definitions

All procedures were performed by 2 experienced endovascular specialists with over 30 years and 15 years of expertise in peripheral artery endovascular interventions, respectively. Technical success was determined by successfully crossing the CTO with a guidewire, either via the true lumen or sub-intimally and it was evaluated by using the procedural records and images. Pre-procedural CTA images were obtained using Revolution HD (General Electric Medical Systems, Waukesha, WI, USA) CT scanner and reviewed using commercial software (VesselIQ, GE Medical Systems). The CT images were compared with the digital subtraction angiography data. Pre-procedural images were reviewed to identify and describe lesion characteristics, such as a tapered proximal or distal cap, which was characterized by a ‘V’ or ‘U’ shape. Flush occlusion was defined as a cap displaying a flat shape, with or without a sloppy morphology, or a reverse ‘U’ shape. Proximal or distal side branches were identified as branches whose orifice were located at the level of the proximal or distal cap, respectively, in which the guidewire could easily slip through. Bridging collaterals were identified as slender vessels located alongside the occlusion, serving as connectors between the non-occluded proximal and distal ends of the segment. Occlusion length was assessed from the proximal to the distal cap of the lesion.

The calcification burden of the occluded vessel was assessed by segmenting the whole lesion in 3D Slicer (slicer.org) and segmentation of calcifications by means of thresholding. The total volume of the CTO (Vol_total_) and the volume of the calcified part (Vol_calcification_) were calculated and the calcification percentage was calculated as ${\mathrm{Percentage}}_{\mathrm{calcification}}=\frac{{\mathrm{Vol}}_{\mathrm{calcification}}}{{\mathrm{Vol}}_{\mathrm{total}}}\times 100$.

The target lesions were primarily managed via an ipsilateral antegrade approach. In instances where this approach was unsuccessful, an alternative strategy involving a retrograde pedal approach was considered and implemented.

### Assessment of outcome predictors and score development

Predictors determining technical success were assessed using logistic regression. Predictors included age >50, the presence of a flush occlusion, the presence of proximal or distal side branches, calcification percentage of the lesion >10%, the presence of a tapered proximal cap, the presence of bridging collaterals, and the length of the occlusion. Univariable logistic regression was used to assess the significance of all the available predictors for the success of the treatment in cohort A (*n* = 59). A multivariable logistic regression model using significant predictors (*P* < .05) in univariable analysis was built with the success or failure of the procedure as an outcome. A predictive score was built as previously described.^8^ For example, factors with *P* < 0.1 identified from the multivariable logistic regression model (cohort A) were used as score variables. Scores were assigned to each predictor based on the odds ratio of the predictor in multivariable analysis to build a final scoring system. The predictive capacity of the score was assessed on an independent set of patients (cohort B [*n* = 25]) collected over a different time frame (∼3 years) to avoid biases introduced by the random selection of test cases. An optimal score threshold was selected based on the receiver operating characteristics (ROC) curve analysis.

### Statistical analysis

Continuous variables were expressed as mean±SD, whereas categorical variables were expressed as frequencies. For logistic regression analysis, odds ratios with the respective 95% CIs were calculated for all included variables, and goodness of fit was assessed with the use of the Hosmer-Lemeshow test. Receiver operating characteristics curves were used to assess the performance of the score in predicting the success of endovascular crossing. The optimal threshold for the score was defined as the ROC curve point where both sensitivity and specificity are maximized. Accuracy, sensitivity, specificity, and area under the curve (AUC) were reported at the optimal score threshold. Statistical analysis was performed with the use of SPSS v. 29 and significance was defined with *P* ≤ .05.

## Results

### Patient characteristics

Patient occlusions were found in 40 left and 44 right lower limbs with a mean patient age of 67.69 ± 10.9 years. The majority of occlusions (*n* = 71) were found at the level of the SFA, 22 of which extended distally to the POPA, and one of them presented as flush occlusion at the origin of the SFA. Thirteen occlusions involved only the POPA. Fifty-six out of 84 occlusions had a tapered proximal cap, 57 had a proximal and 27 had a distal side branch, whereas 37 occlusions had bridging collaterals. Analysis of calcification showed that in 54/84 (64.3%) more than 10% of the lesion volume was calcified. In 26 out of 84 patients (30.95%), the procedure failed due to inability to cross the lesion with the guidewire.

### Score construction

Univariate analysis indicated that all examined variables were significant apart from the length of the occlusion and the presence of bridging collaterals. Multivariate logistic regression including all variables identified in univariate analysis, indicated a series of factors with *P* < .1 eligible for inclusion in the score. These included age > 50 (*P* = .036, odds ratio (OR) 53.7 with 95% CI, 1.284-2245), calcification percentage of the occlusion >10% (*P* = .011, OR 16.63 with 95% CI, 1.899-145.654), the presence of a flush occlusion (*P* = 0.02, OR 15.564 with 95% CI, 1.531-158.204), the presence of a distal side branch (*P* = 0.018, OR 9.879 with 95% CI, 1.493-65.377), and the presence of a proximal side branch (*P* = 0.064, OR 23.369 with 95% CI, 0.831-657.218). Relative to their OR, the factors were assigned the following EndoVAscular CROSsing Score for Chronic Total Occlusion (EVACROSS-CTO) scores: +10 points for age > 50 years, +3 points for a flush occlusion, +4 points for a proximal, and +2 points for a distal side branch, as well as +3 points for calcification of the CTO of >10%. The maximum achievable score is 22 points (Tables 1 and 2).

**Table 1.** Multivariable logistic regression results, statistically significant values (*P* < 0.05) are shown in bold.

Variable	P	OR
**Age >50**	**.036**	53.700
**Flush occlusion**	**.020**	15.564
**Distal side branch**	**.018**	9.879
**Calcifications >10%**	**.011**	16.633
**Proximal side branch**	**.064**	23.369
**Tapered proximal cap**	.593	.545

**Table 2.** EVACROSS-CTO factors and score points based on multivariate logistic regression results.

EVACROSS-CTO	
**Score factor**	**Points**
**Age >50**	+10
**Flush occlusion**	+3
**Distal side branch**	+2
**Calcifications >10%**	+3
**Proximal side branch**	+4
**Total**	22

### Score assessment

**Figure 1.**
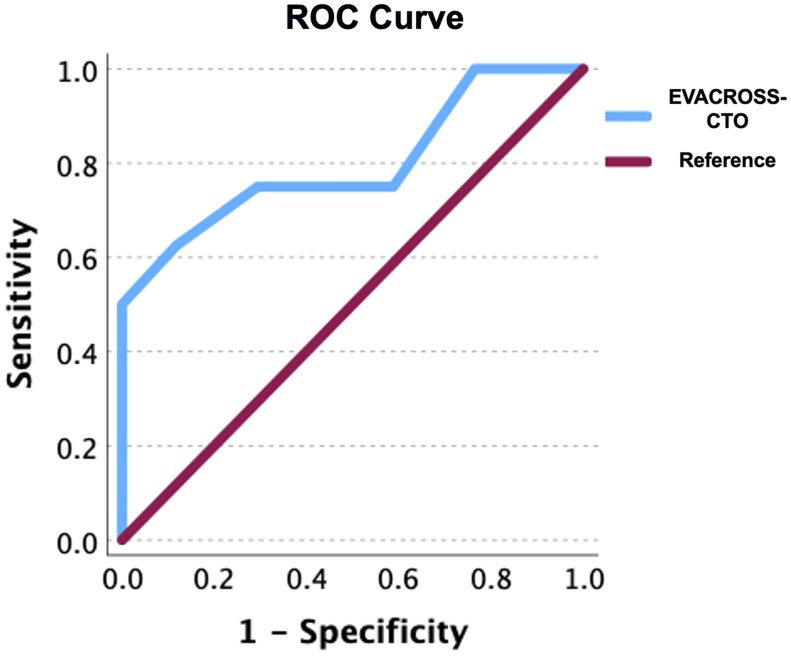
Receiver operating characteristics (ROC) curve of the EVACROSS-CTO evaluation.

**Figure 2.**
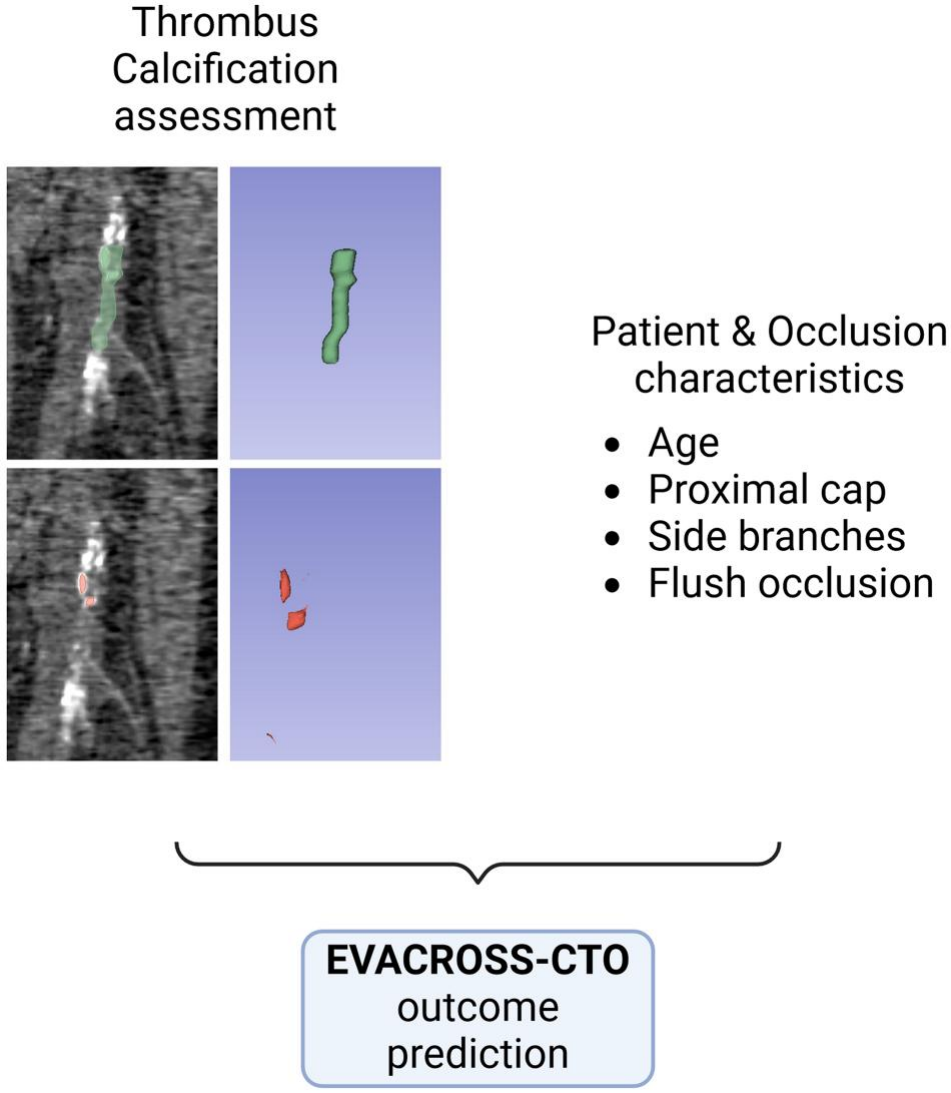
Flow chart outlining the process for the calculation of the pre-procedural EVACROSS-CTO.

Patients of cohort B were scored based on the EVACROSS-CTO score and an ROC curve was created based on the scores and the respective outcome. EVACROSS-CTO was able to predict the success of endovascular crossing with an AUC-ROC of 79.8% (95% CI, 58.5%-100%). A score >16 yielded a sensitivity of 75% with a specificity of 70.6% in cohort B (Figure 1 and Table 3). The procedure to calculate the score prior to the procedure is outlined in Figure 2.

**Table 3.** Receiver operating characteristics (ROC) curve coordinates used to determine the optimal threshold (red font) for the respective sensitivity and specificity values.

Positive if greater than or equal to	Sensitivity	1 − Specificity	Specificity
−1.0000	1.000	1.000	0
5.0000	1.000	0.941	0.059
11.0000	1.000	0.824	0.176
12.5000	1.000	0.765	0.235
13.5000	0.750	0.588	0.412
14.5000	0.750	0.471	0.529
15.5000	0.750	0.353	0.647
**16.5000**	**0.750**	**0.294**	**0.706**
18.0000	0.625	0.118	0.882
20.0000	0.500	0	1
21.5000	0.375	0	1
23.0000	0.000	0	1

## Discussion

Herein, various determinants affecting the success of recanalization procedures for peripheral artery CTOs, particularly concerning lesions in the SFA and POPA, have been identified. These determinants were observed in pre-procedural CT angiography and correlated with the successful or unsuccessful guidewire crossing of the CTOs. Subsequently, these factors were utilized in developing the EVACROSS-CTO score, designed to predict the likelihood of a successful EVT outcome of a CTO in one or a combination of the aforementioned arterial segments. After conducting logistic regression, 5 key variables were identified: age over 50, flush occlusion, proximal side branch, distal side branch, and calcification burden over 10%.

Treatment of CTO in coronary and peripheral arteries is technically challenging with a high failure rate ranging between 20% and 40%.^1^^,^^9^ Technical success in the current study is in line with the literature. The demand for improved patient selection and treatment planning has led to the development of new scoring algorithms.^1^^,^^3^^,^^7–10^ Recent studies^3^^,^^11^^,^^12^ employed a variety of demographic, clinical, and anatomical variables to predict the success of peripheral artery CTO interventions. Common variables identified included patient age, diabetes, and history of previous revascularization. Anatomical factors such as the length and severity of occlusion, degree of calcification, morphological classifications, and specific lesion locations (eg, proximal superficial femoral artery and distal POPA) were critical in their predictive models. These studies demonstrated that their scoring systems provided high predictive accuracy, with an AUC ranging between 0.82 and 0.87. This emphasizes the effectiveness of a multifactorial approach in developing predictive scores for peripheral artery CTO interventions. Notably, the abovementioned scores have been predominantly directed towards addressing lesions in BTK and coronary arteries. The current study is dedicated to establishing a CTO scoring system, specifically tailored for femoropopliteal lesions, known for their frequent incidence with significant length and high calcification burden. In contrast to prior research endeavours, scoring in this study relied on pre-procedural CTA scans, an imaging method previously validated for treatment planning and prognostication.^13^^,^^14^ The selection of determinants was primarily guided by previous studies,^2^^,^^3^^,^^7^^,^^15^ supplemented by observations made during interventions, such as the propensity of the guidewire to traverse into a side branch, particularly in conjunction with a flush occlusion. The study yielded a predictive accuracy of 79.8%, which is a good performance given the multiple factors affecting the score. Notably, the inclusion of a substantial number of patients with lengthy occlusions did not show a significant impact on the success of the interventions, consistent with findings from prior research. In contrast, studies focusing on BTK CTO lesions have suggested that lesions exceeding 200 mm may influence procedural success.^3^^,^^7^ This discrepancy underscores the potential variations in CTO characteristics based on their specific vascular locations.

To ensure the EVACROSS-CTO score is of practical clinical use, its primary utility could potentially focus on prioritizing patients for angioplasty. By incorporating variables such as patient age, occlusion characteristics, and calcification burden, the score effectively identifies those most likely to benefit from the procedure. This prioritization not only enhances patient selection by minimizing unnecessary interventions for those with lower probabilities of success but also optimizes resource allocation within the healthcare system. Additionally, the physician would be equipped to preemptively identify scenarios where access might encounter a higher failure rate and could prepare with available devices to improve efficacy. Furthermore, this scoring system might additionally assist operators in determining the level of effort required to achieve technical success. Finally, patients could receive precise information regarding the complexity of their CTO and the probability of a favourable treatment outcome. Despite the valuable insights, there are notable limitations. Such scores can be complex, relying on comprehensive and subjective assessments, which may impact reproducibility and consistency across different clinical settings. The predictive accuracy of these models can vary based on the patient population and CTO characteristics, making them less universally applicable. The need for thorough validation in diverse populations remains a challenge to ensure the broad utility and reliability of these scoring tools. The study has several potential limitations that need consideration. Firstly, it is an observational, single-centre, retrospective study, which might limit the generalizability of the findings to broader populations. However, conducting this scoring system in various centres could verify its accuracy and applicability across different settings. Secondly, despite including a considerable number of patients with CTOs, the final sample size might still be relatively limited. Moreover, although the EVACROSS-CTO score demonstrated predictive capacity in cohort B, its ability to independently guide clinical decision-making might be limited. Further validation in larger and more diverse cohorts is essential to assess its reliability and applicability across different patient populations and clinical settings. Additionally, the score was specifically developed using data from patients with atherosclerotic PAD. Its relevance to other conditions, such as Berger’s disease or post-embolic and thrombotic occlusions, remains unclear, as these conditions were not represented in the study population. Given the distinct pathophysiological characteristics of these diseases, the score’s applicability to such cases may be limited.

Future versions of the EVACROSS-CTO score could be refined to include factors that predict potential procedural complications, such as vessel perforation or distal embolization. This would not only increase its clinical value but also help provide a more holistic approach to decision-making. Additionally, validating the score in broader patient populations, including those with conditions like thrombotic occlusions, could significantly enhance its applicability and relevance across diverse clinical scenarios.

## Conclusion

The development and validation of the EVACROSS-CTO score represents a significant step forward in predicting the success of EVT for CTOs in femoropopliteal arteries. This predictive tool incorporates variables derived from pre-procedural CT angiography and demonstrates promising predictive capacity in determining procedural outcomes. Further validation of the applicability of this score across diverse populations and clinical settings is needed in order to confirm its reliability and broader utility. Despite these considerations, the use of this scoring system holds considerable potential in aiding clinicians to make informed decisions and optimize patient care strategies in managing femoropopliteal CTOs.
